# Ukrainian Refugees and Welfare Deservingness: A Comparative Study of UK Government Discussions Around the 2022 Ukraine Conflict and 2015 Migrant Crisis

**DOI:** 10.1111/1468-4446.13219

**Published:** 2025-04-27

**Authors:** Joshua Garland, Juhyun Lee

**Affiliations:** ^1^ Centre for Sustainability Studies Lund University Lund Sweden; ^2^ Department of Social and Political Sciences University of Milan Milan Italy

**Keywords:** migrant crisis, racialisation, refugees, Ukraine, United Kingdom, welfare deservingness

## Abstract

Recent years witnessed mass migration towards Europe, from Russia's 2022 invasion of Ukraine and the 2015 Migrant Crisis linked to war in Syria. This article explores UK government discussion around these two significant crises, focussing on the challenges they present and the portrayal of refugees. It asks how far ministers' language differentiated between Ukrainians and Syrians regarding welfare deservingness. Thematically analysing over 100 official speeches, statements and press releases, the extent of racialisation and welfare chauvinism in ministers' discourse on refugees is revealed. Clear racialisation was found between the two refugee groups, but welfare chauvinism persisted for Ukrainians despite more favourable language, reflecting continued conditionality within UK government discussions of migration phenomena that may hold long‐term implications for Ukrainian refugees in the UK.

## 1 Introduction

Conflicts have resulted in millions of people becoming displaced and seeking safety outside their home countries. Conflict contributed to the 2015 Migrant Crisis in which hundreds of thousands of Syrians reached European borders, including the UK. War also drove millions of Ukrainians to leave their homes following Russia's 2022 invasion.

While recent studies focus on public refugee perceptions (Hedegaard and Larsen 2022; Moise et al. 2024; Nielsen et al. 2020), this timely article seeks to understand UK government responses through press releases, statements and speeches regarding these crises, addressing a current dearth of research on Ukrainian refugees. It asks why ministers' language differed between Ukrainians and Syrians and how this can be helpfully related to a wider social scientific concept of welfare deservingness, asking: *How is welfare deservingness and racialisation reflected in UK government language around Ukrainian and Syrian refugees*? Welfare deservingness, chauvinism and racialisation literatures form this article's conceptual basis.

A thematic analysis of official UK government communications was used to identify central trends in minister portrayals of arrivals, examining convergence and divergence in attitudes towards Ukrainians and Syrians comparatively. Clear racialisation and deservingness perception‐related distinctions were found with positive and negative language towards Ukrainians and Syrians respectively. Nonetheless, benefit conditionality was observed more for Ukrainians. Consequently, a longer‐term integration perspective is required in the UK since racialisation and welfare chauvinism could draw attention away from domestic challenges, like long‐term growth encompassing improving labour shortage in certain areas, which refugee integration could support.

## 2 Background

### 2.1 Invasion of Ukraine

Russia launched a large‐scale invasion of Ukraine on 24 February 2022 following a wider history of Russia‐linked aggression against Ukraine, including the 2014 annexation of Crimea (Ukraine Ministry of Foreign Affairs 2022; Walker 2022). Russia encountered staunch Ukrainian resistance and evidence of war crimes has emerged (Internation al Criminal Court 2022). A mass displacement of Ukrainians has consequently been witnessed within and beyond the country's borders, including to the UK. By mid‐September 2022 over seven million people left Ukraine, peaking in the first month following invasion (UNHCR 2022a, 2022b).

These were almost all women and Ukrainian (86% and 99%). Many (55%) were aged between 35 and 59 and 47% university educated (UNHCR 2022a). UN‐provided data indicated that three‐quarters left Ukraine with others; almost always family members (91%) of whom 36% were under 18. UK‐specific statistics reflect this dominance of women (70%), with 61% aged 19–64. The number of under‐18s appeared congruent with UN‐compiled data, comprising 33% of the more than 80,000 Ukrainians arriving in the UK before July (Home Office 2022). These demographics are crucial to understand the language used for Ukrainian refugees in concert with deservingness and its intersections with family and sex through need and control criteria (Heuer and Zimmermann 2020; Nielsen et al. 2020).

Research into Ukrainian refugees remains limited and represents this article's key contribution. Of existing studies, responses were found to vary notably between countries (Kaim et al. 2024; Letki et al. 2024; Luděk et al. 2023), including societal resilience concerns regarding host nations' self‐perceived capacities to cope with migration‐related pressures (Kaim et al. 2024). However, unlike the present study, these have focused on surveying public attitudes and not government ministers that are central to issue framing and policy responses, and nor have they focused on the UK.

### 2.2 Migrant Crisis

2015 saw another large‐scale displacement of people, known as the Migrant Crisis. By 2015's end, almost one million individuals entered the EU—UK included—after fleeing war and persecution (Spindler 2015; UNHCR and UNFPA and WRC 2016). This was reflected by significant increases in illegal border crossings and asylum applications (Frontex 2015). Arrivals largely originated from Syria (50%) and other MENA countries (Frontex 2015). Syria had experienced civil war since 2011 coupled with the rise of the Islamist terror group, ISIL.

Among arrivals, one‐quarter were children, 17% women and 58% men (UNHCR 2016; UNHCR and UNFPA and WRC 2016). Data reflecting 2015's first 6 months reported a higher proportion of men with most Arab and Sunni Muslim (83%, 78% and 87%, respectively) (UNHCR 2015). Seventy one per cent were aged between 18 and 35 and 40% university educated (UNHCR 2015). This demographic profile differed to Ukrainians', although Ukrainian men's military conscription accounts for their lower representation and was a leading cause behind family separations (UNHCR 2022a).

While fleeing war—a ‘deserving’ reason (Hedegaard and Larsen 2022)—this is argued as insufficient to understand reactions to refugees; including suggestions that the wars' geographic proximities led to different feelings of involvement and more Ukrainian refugee acceptance (Moise et al. 2024). Instead, cultural proximity well‐captured through the mixed embeddedness concept, intertwined with racialisation and anti‐Muslim bigotry, are suggested to provide more convincing accounts. This conceptual basis is now developed.

## 3 Literature

Welfare chauvinism refers to ideas that welfare benefits should be predominantly reserved for natives, linking into debates around whether migrants deserve benefits based on their societal contribution, reflecting CARIN criteria introduced below (Landini 2022). Chauvinism has been rising in the UK alongside right‐wing party support, and state support for migrants increased in issue salience during the Migrant Crisis (Hedegaard and Larsen 2022; Nielsen et al. 2020). Russia's Ukraine invasion caused another significant refugee influx into the UK, yet the language used for Ukrainians and Syrians differed despite this broader UK context.

This article investigates how positive language towards Ukrainian refugees was present alongside anti‐immigrant sentiment in the UK, including towards Eastern Europeans (Landini 2022; Schweyher et al. 2019). It casts light upon whether this reflects racialisation related to Syrians' cultural and socio‐economic differences and whether it indicates a partial overcoming of welfare chauvinism. Literature is therefore organised into two streams concerning racialisation, and welfare chauvinism related to deservingness criteria.

### 3.1 Racialisation and Anti‐Muslim Bigotry

Racialisation is defined as a system of socially‐constructed categories based on physical characteristics associated with ethnicity (Roth et al. 2023). These become linked to socio‐political and/or economic concerns that further attach to different cultural and religious practices (Jackson 2018; Younis and Jadhav 2020). This process creates an ‘Other’ with an identity unlike one's own, resulting in phenomena like anti‐Muslim bigotry where fears and prejudices become ascribed to Muslims (Jackson 2018).

The UK has long been a secular state with a historically colonial and Christian identity (Jackson 2018; Poynting and Mason 2007). Resultantly, a distinction between religious and political practice exists alongside an Othering of non‐Christians and non‐Whites, including Muslims (Jackson 2018; Poynting and Mason 2007), coupled with racial superiority–inferiority hierarchies that supported colonialism (Fanon 1986; Goldberg 2002). This history informed post‐war migration flows when former colonial subjects increasingly immigrated to the UK (Poynting and Mason 2007), including Caribbean migrants' 1948 arrival onboard Windrush, with the UK shifting to a net migration country where benefits are shared across different ethnic groups from mid‐1980 (Lindert 2014; Sturge 2024). This trend continued with increased EU migration following the 2004 EU accession of CEE countries like Poland. Since the 2016 Brexit vote EU migration has reduced while non‐EU migration continues to increase (Sturge 2024). These trends have been subject to racial tensions, reflected by 1981 riots and political statements around integration, noted later. Muslim communities were also established with most UK Muslim's heritage traceable to South Asia (Jackson 2018; Poynting and Mason 2007). Their arrival was similarly coupled with anti‐immigration sentiments (Poynting and Mason 2007).

2001 marked a key year for racialisation and anti‐Muslim bigotry in the UK, following social unrest by young Muslims in England involving police clashes and property damage (Jackson 2018; Poynting and Mason 2007). This followed 1980s anti‐blasphemy book‐burning protests against Rushdie's *The Satanic Verses* on UK streets (Poynting and Mason 2007). Through these events, Islam and the UK's Muslim population became associated more with social cohesion‐related fears as a socio‐political challenge (Alba and Foner 2015; Carmon 1996; Goldberg 2009).

Muslims were cast as a threat to ‘British’ social identity and values like democracy, freedom of expression and secularism, pointing towards perceived cultural tensions seen to undermine UK norms and socio‐political systems (Jackson 2018; also, Goldberg 2009; Hedegaard and Larsen 2022; Moise et al. 2024). This resulted in a ‘good’–‘bad’ Muslim distinction in political, media and public rhetoric, related to integration and national value questions (Jackson 2018; Younis and Jadhav 2020). This dualistic distinction exists also in security threat construction through counter‐terrorism programmes like PREVENT; a 2007 anti‐radicalisation reporting policy for public bodies (Jackson 2018; Younis and Jadhav 2020). PREVENT was argued to racialize Muslims as the group most susceptible to radicalisation and subsequently a key national security threat (Rodrigo‐Jusué 2023), resulting in discriminatory public sector provision (Younis and Jadhav 2020).

This was reinforced through UK‐targeted Islamist terror attacks that internalised Muslim threat perceptions where historically this Other existed externally, juxtaposed to a Western, Christian identity (Jackson 2018; Rodrigo‐Jusué 2023). This externality includes modern UK wars in Muslim‐majority Iraq and Afghanistan (Martin 2017; Poynting and Mason 2007). Domestic events include the 2005 7/7 bombings, the 2013 murder of off‐duty soldier Lee Rigby and 2019's London Bridge attack. Islamist terrorists with Syrian links also conducted attacks on the Continent, including Paris in November 2015.

Muslim identity and practice, including religious norms, thereby formed sites for threat perception through racialisation processes whereby Muslims become associated with terrorism or social tensions through sharing a certain culture. Anti‐Muslim bigotry as a form of cultural racism results (Jackson 2018). This is additionally demonstrated by conspiracy theories about UK Islamification through Muslim population growth, mosque construction and Sharia law enforcement (Jackson 2018; Martin 2017; Swami et al. 2017). This could be seen as an extension of anti‐migrant discourse in the UK that accompanied shifts to net emigration. An infamous example is Conservative politician Enoch Powell's 1968 ‘Rivers of Blood’ speech warning against the threat of non‐integration and a suggested imposition of migrants' own origin country and religious norms onto wider society. Recently, the 2016 ‘Breaking Point’ poster drew directly upon 2015 Migrant Crisis experiences, depicting the arrival of non‐White refugee men portrayed as unwanted burdens and national threats, during Brexit Referendum campaigning.

It was also reflected in increased support for far‐right and/or anti‐immigrant political parties (Martin 2017; Poynting and Mason 2007; Swami et al. 2017). A 2–3 times higher support for populist parties was found in the UK in 2015 compared to 2000, with similar occurring in other European countries sharing historic identity construction against a Muslim Other with fears around values like freedom of expression (Jackson 2018; Milanovic 2016). Accordingly, this lower preference for Muslims and related bigotry can be clearly witnessed in labour market outcomes across Western Europe. A recent study showed that MENA individuals experienced a double penalty involving low employment and low job quality more than Eastern Europeans or other ethnicities in 16 Western European countries, UK included (Lee 2024). Moreover, negative perceptions towards refugees were found concerning linkages between work as contribution to the host society and welfare deservingness in the UK, with unemployment possibly used to engage in anti‐refugee ‘dog‐whistle’ politics (Calo et al. 2024). This was coupled with policies discussed as placing barriers to non‐native individuals' labour market integration, similarly to Lee (2024).

Mixed embeddedness is important here, concerning immigrants' assimilation to destination country culture which can inform natives' perceptions towards arrivals (Kloosterman et al. 1999). Under this view, immigrants likely collide with two cultural backgrounds. Those retaining origin cultures are often less integrated in receiving countries. Therefore, less‐integrated migrants following strong origin norms like Muslims are associated with both their own cultural practices under mixed embeddedness, and host society's negative perspectives based on anti‐Muslim bigotry (Goldberg 2009; Gracia et al. 2016; Jackson 2018; Kloosterman et al. 1999).

Beyond culture and ethnicity, origin countries' economic development could additionally shape refugee stereotypes. This is because developed economies' citizenship premiums continue since push–pull factors between global North and South have not reduced. Accordingly, borders are favourably open to immigrants holding high human or material capital rather than those less educated or with lower economic resources (Ellermann 2020; Milanovic 2016); leading to racialisation based on ethnicity‐based identity markers, religion and economic considerations (Goldberg 2009).

Arrivals may therefore be discussed with a different lexicon by UK ministers regarding how they are perceived to be able or willing to culturally integrate while contributing to society; supporting themselves through human and material capital. Racialised as security and social threats, Syrians may be discussed in less preferable terms than Ukrainians.

### 3.2 Welfare Chauvinism and Deservingness

Within welfare states, institutionally‐distinguished insiders and outsiders benefitting under social security and labour market systems exist. However, increasing arrival numbers create questions about different groups' benefit deservingness, revealing welfare chauvinism (Eger et al. 2020; Reeskens and van der Meer 2019). Hence, ‘immigrants’ were found with least deservingness among categories including ‘family’, ‘women’, ‘children’ and ‘elderly’ (Heuer and Zimmermann 2020; Nielsen et al. 2020). Relevantly, within social policy research Van Oorschot (2000) defined deservingness criteria including *control*, *attitude*, *reciprocity*, *identity* and *need* (CARIN) that have been used to understand how natives regard immigrants as deserving benefit recipients (Table 1) (Meuleman et al. 2017; Nielsen et al. 2020). Although results vary across European countries according to different welfare systems, CARIN revealed the importance of specific immigrant characteristics informing deservingness (Eick and Larsen 2022; Heuer and Zimmermann 2020).

**TABLE 1:** CARIN applied to refugee perspectives.

Criterion	Characteristics	Expected deservingness
Control	Capacities to influence and responsibility for current situations (as cause, solution). Fulfilment of related duties. Link to demographics (sex, age).	Women and vulnerable groups, including children, the elderly and families as less able to directly address situations (such as conflict) and more deserving.
Attitude	(Good) behaviour and gratefulness for support received.	Arrivals meeting behavioural expectations (normative, legal) in host societies could have higher deservingness. Illegal arrival means may reduce deservingness accordingly.
Reciprocity	Contribution to host society in the present through taxes and/or labour market participation.	Ability to directly enter into work, contributing to the host country without receiving additional financial support, as increasing deservingness.
Identity	Assimilation extent, cultural proximity and us‐them distinctions. Mixed embeddedness.	Greater assimilation expected to increase deservingness. This may relate to pre‐existing cultural similarities between natives and arrivals, natives' bigotry against arrivals on religious or similar grounds and the extent arrivals retain origin country norms.
Need	Limited (material) resources. Linked to age, sex, family units.	Similar to control, women and vulnerable individuals seen to have greater need and thus higher deservingness.
Social investment	Potential for future—not present—contribution to host society. Requirement for education and training beforehand.	Arrivals perceived to require host country support and investment in education and training before contributions possible, reducing deservingness.

Van Oorschot's non‐mutually exclusive categories have commonly been applied to study public immigration perceptions (Landini 2022), but it is argued here that they can serve as a useful lens to examine different aspects of political actors' portrayal of migration and refugee phenomena. Others indicated how political actors' depictions of arrivals influences public opinion and media coverage, with language used purposively within electoral strategies to reduce blame for welfare pressures through linkages to immigration (Abbas and Chrisp 2023; Rodrigo‐Jusué 2023). This includes deservingness, framed through CARIN (Baekgaard et al. 2023; Esmark and Schoop 2017). In the UK, this politician language and public opinion of welfare deservingness association was seen in how people's support for benefit payments and eligibility reduced following negative political‐level constructions of claimants (Jensen and Kevins 2019).

## 4 Research Question

The research question posed is: *How is welfare deservingness and racialisation reflected in UK government language around Ukrainian and Syrian refugees*? This is investigated through multiple themes reflecting CARIN. Firstly, the *identity‐attitude* relationship.

It could be expected that 2015 arrivals, being predominantly Muslim and arriving through illegal means, could be seen negatively by natives. This can reflect natives' pre‐existing anti‐Muslim bigotry rooted in perceptions of Islam and experiences of Islamist terror, alongside mixed embeddedness in which Muslim arrivals maintain their cultural and religious norms, practices and dress. White Ukrainians arriving through legal channels with a shared Christian heritage may therefore be welcomed more within UK ministers' language (Baekgaard et al. 2023; Landini 2022; Nielsen et al. 2020).

*Control* and *need* may also be observable through demographic descriptions of arrivals (Kootstra 2016), recalling how women and families are considered more deserving than men who might be expected to take greater responsibilities at home, or have higher material resources and thus less need (Freedman et al. 2017; Mesarič and Vacchelli 2021; Nielsen et al. 2020; Plümper and Neumayer 2021; Reeskens and Van Oorschot 2012; Rettberg and Gajjala 2016; Van Oorschot 2000). Ukrainian men underwent conscription with expectations that they fulfil conflict‐related responsibilities and duties. To recall, in 2015 only 17% and 25% of refugees were women and children respectively (UNHCR 2016). In 2022, 70% were women (UNHCR 2022a). Ukrainians may therefore be more positively depicted as deserving.

Related to origin country wealth and anti‐Muslim bigotry (Heuer and Zimmermann 2020), Ukrainians might be portrayed as being more deserving based on perceived abilities to enter the UK's workforce and contribute to society in the present, including through taxation (Landini 2022). Syrians may be discussed more regarding educational and training investment needs. The former speaks to *reciprocity*, the latter to *social investment* as possible future contributions only after additional host country expenditure (Heuer and Zimmermann 2020).

## 5 Methods

Gov.uk, Hansard (Commons and Lords debates) and Parliament's website (written answers and statements) were consulted under a content analysis approach (Esmark and Schoop 2017). As reliable primary sources (Rodrigo‐Jusué 2023), these were chosen since they represent core means through which UK authorities communicate their activities, policy platforms, minister speeches and crisis reactions. They provided windows into official business and discussion (Huysmans and Buonfino 2008; Landini 2022), yet are not often fully utilised (Rodrigo‐Jusué 2023).

These sources were therefore well‐suited to investigate ministers' perspectives concerning the Migrant Crisis and Russia–Ukraine war displacement. Certainly, discussions and language surrounding refugees, migrants and asylum‐seekers have repercussions on the identities imposed upon them and their treatment in practice (Grove and Zwi 2006). It is therefore important to know ministers' language to understand these processes, including since they can inform wider public discussion (Baekgaard et al. 2023). Ministerial language and not specific policies were subject to analysis. Ethically, collected documentation reported only the language and points expressed by public‐facing government ministers, including the Prime Minister, so no private individuals were involved or identifiable. Data was aggregated and rendered anonymous, while secured on an author‐specific password‐protected computer.

Data collection search terms were ‘migrant’ or ‘refugee’ and ‘Syria’ or ‘Syrian’ for 2015, or ‘Ukraine’ or ‘Ukrainian’ for 2022. Used in conjunction with each other, keywords were later searched for separately for comprehensiveness. The timeframe was 1–20 September 2015, capturing numerous key Migrant Crisis events. This included UK government policy announcements like the expansion of its Vulnerable Persons Resettlement Scheme (VPRS) to bring 20,000 Syrians to the UK on 7 September. This came only 5 days after Alan Kurdi's body was found on 2 September with photographs of this incident gaining widespread media coverage, becoming an iconic image humanising the Migrant Crisis for many and leading to more welcoming policy shifts in countries including Canada (Bodrunin 2018; Hellmueller and Zhang 2019; Kingsley and Timur 2015; Spindler 2015). Many EU states nonetheless closed their borders and introduced more entry restrictions from 15 September, responding to increasing refugee movements within Europe (UNHCR 2015, 2016; Spindler 2015), with the time after the Kurdi photograph also witnessing increased border security frames in German media (Hellmueller and Zhang 2019).

For Ukraine, the timeframe extended from the conflict's start on 24 February to 15 March 2022; an equivalent 20‐day period. This captured when significant numbers of Ukrainians were displaced and notable issue‐attention was afforded refugee movements by news media (UNHCR 2022a). UN figures reported three million border crossings from Ukraine in this period (UNHCR 2022b), exceeding arrival numbers seen by the end of 2015. While time difference between 2015 and 2022 could have influenced minister language, the UK government remained Conservative‐led and observed differences could usefully be understood through the CARIN framework, making an acceptable basis for comparison of groups from different times.

*N* = 357 press releases, official statements and speeches were collected and manually checked for relevance to the refugee theme. Where missing, documents were removed. Consequently, *n* = 171 documents were retained for analysis (48% of those originally collected), with 63 for 2015 and 108 for 2022. This sample represented all available materials from the sources for the specified timeframes.

A thematic analysis involving iterative code development through a pilot phase, elaborated below, was conducted by authors with experience of this method. Codes defined by the pilot were subsequently applied to the full dataset and resulted in key theme identification later summarised through quantification. This revealed trends in UK government ministers' language use (Braun and Clarke 2006; Vaismoradi et al. 2016). Themes reflected not only the most frequent perspectives, but also those directly relevant to the research question and related literature debates (Braun and Clarke 2006). Beginning with open coding, relevant words, terms and phrases were recorded then evaluated for shared ideas and topics before being grouped (Krause and Bucy 2018). Code groups and thematic consistency was sought across case years.

Reliability was supported through the pilot coding of 14% of the document bank (24 documents, 12 per year) which was systematically sampled for equal representation across years and sources. A coding schema was devised using this pilot and subsequently applied to the whole document bank, as noted. CARIN criteria guided this alongside three considerations: what causes underlying refugee flows were identified? How were ministers' responses framed? How were refugees portrayed?

In this way, the theory supported theme identification while familiarity with the data was gained through the pilot which in turn enabled a clear definition of each code grounded directly in the data itself (Schreier 2019). Although intercoder reliability statistics are not applicable in cases where a single researcher led code development and application, the clear definition of codes that are also based in a familiarity with the data, its content and meaning, are key aspects of content analysis reliability that were satisfied here (Boydstun 2023; Byrne 2021; Schreier 2019). Table 2 presents these codes and example statements from the document bank by which they were identified and recorded during the coding process.

**TABLE 2:** Codes and matched statements.

Code	Statements
Suffered/suffering	‘reduce the suffering’, ‘the plight of people in desperate need’, ‘people who have been affected so devastatingly’, ‘in their hour of need’
Illegitimate/undeserving	‘those who do not have a genuine claim’, ‘those with no right to be in the UK’, ‘in the country illegally’
Legitimate/lawful	‘eligible to come’, ‘living lawfully in the UK’, ‘offering safety to those who need it’, ‘in need of protection’
Exploited	‘exploiting these people’, ‘exploitation’
To return to home country	‘do not want to be refugees’, ‘want to be Ukrainians living in Ukraine’, ‘want to be able to go back and rebuild’, ‘until they are able to return’
Refugee	‘many are refugees’
Migrant	‘some are economic migrants’, ‘where the migrants are coming from’
Victim	‘they are victims’
War	‘continued its war’, ‘terrible, unjust war’
Terror	‘ISIL’, ‘extremist Islamist terrorism’
Violence	‘killing civilians’, ‘savage, indiscriminate, unprovoked aggression’
Smuggling/trafficking	‘target trafficking routes’, ‘detect and disrupt the smuggling gangs’
Pressure (domestic)	‘relieving the huge pressure’, ‘stresses and strains’, ‘burden on local communities’, ‘capacity issues’
Socio‐political challenge	‘social cohesion’, ‘that issue of integration’
Economic challenge	‘economic hardship’, ‘economic support’
Education and training	‘schools’, ‘training’, ‘education’, ‘skills’
Employment	‘entitlement to work’, ‘employment’, ‘access to the labour market’, ‘take jobs in Britain’
Welfare	‘full and unrestricted access to benefits’, ‘our welfare system’

Coding focused on the main text, excluding (sub‐)titles which repeated main text content, and code occurrence frequency was recorded on aggregate across the documentation before being examined for commonalities. It was these for which frequencies were recorded by year and all coding was systematically conducted and structured using NVivo 14. Codes and umbrella themes are relayed throughout the below and summarised numerically.

## 6 Results

### 6.1 Identity and Attitude

**FIGURE 1:**
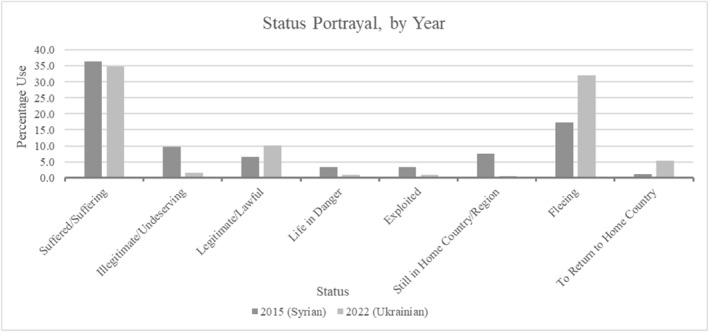
Arrivals' status portrayals.

Identities and attitudes are key criteria influencing arrivals' perceived deservingness. The former relates to anti‐Muslim bigotry and mixed embeddedness (assimilation). The latter concerns good behaviour linked to legality and both emerged from the data (Figure 1). Although Syrians were described as suffering like Ukrainians, there was greater use of language linked to illegitimacy and exploitation. Contrastingly, Ukrainians were portrayed as more legitimate and fleeing genuine situations. This suggested that while war may be a legitimate migration motive (Nielsen et al. 2020), it is insufficient to explain UK ministers' construction of arrivals' deservingness. Proximity to Ukraine may have led European states, including the UK, to ‘feel involved’ in the conflict and to respond more positively to displacement (Moise et al. 2024, 18). However, cultural and racial stereotyping related to UK secularism and cultural proximity through mixed embeddedness arguably played an important role.

Regarding illegitimacy and exploitation, the constructed Syrian identity related to their arrival by illegal means, including through smuggling gangs. This spoke to the attitude criterion and entry regulations where identity is factored into risk assessments concerning arrivals (Abrahamsen 2005; Amoore 2006; Van Oorschot 2000). Identity extends to places of origin and particularly to less developed or unstable locations portrayed as fostering criminality and/or terrorism. Arrivals from such places can thereby become associated with these issues as threats (Abrahamsen 2005).

Syria's civil war was a primary cause of displacement and GDP decline, coupled with ISIL's rise domestically. Most Syrian arrivals were Muslim men and so racialisation grounded partially upon anti‐Muslim bigotry arguably contributed to negative ministerial language concerning them (Goldberg 2009; Moise et al. 2024; Rodrigo‐Jusué 2023), contrasting against Ukrainian‐related positivity. Recent literature supports this, finding significant deservingness penalties for Muslims compared to Christians in Western Europe (Hedegaard and Larsen 2022; Landini 2022).

Moreover, actual policies regarding Syrian and Ukrainian refugees demonstrated different approaches. For Syrians, there were clear conditions defining eligibility to enter the UK through VPRS alongside a cap of 20,000 people over 5 years, emphasising women, children and those with serious medical conditions (Home Office 2017), with up to 3000 children also targeted through the Vulnerable Children's Resettlement Scheme. No limits to the number of Ukrainians benefiting from three bespoke visa routes, including ‘extension’, ‘family’ and ‘sponsorship’ schemes, nor specific demographic conditions like those for Syrians, were defined (Cuibus et al. 2024). These Ukraine schemes enabled Ukrainians already holding a visa to extend its validity, join UK‐based family members or to stay with volunteer households while permitting access to work and benefits (Home Office 2024). The scrutiny and screening of Syrian refugees through UNHCR (potential applicants) and IOM (medical assessments) cooperation (Home Office 2017), plus general rules to only accept Syrians from inside the region's refugee camps, were reflected in the language of illegitimacy and illegal border crossings found particularly concerning Syrian men.

Mixed embeddedness can additionally be drawn upon regarding deservingness' identity criterion through which Muslims may be understood to challenge the UK's longstanding secular principles through religious practices and lower assimilation (Goldberg 2009; Hedegaard and Larsen 2022). Arrivals may be tolerated more within Western societies when seen as unchallenging and compliant in identity and behaviour (Grayson 2013). Again, identity becomes associated with legitimacy, risk and deservingness. Fears of terrorism's spread beyond Syria and the region may have contributed to this; building an identity tied to Syrian's religion, sex and origin, alongside notions of control and border security relevant to migration phenomena (Abrahamsen 2005; Huysmans and Buonfino 2008).

The idea they broke the law to enter the UK could also contribute to othering processes contrasted against a law‐abiding ‘us’ (Grove and Zwi 2006). The illegitimacy within minister discussions around Syrians therefore linked again into deservingness with attitude criteria reflecting arrivals' good behaviour. This could be challenged when associated with crime and/or illegal border crossings, reducing deservingness (Van Oorschot 2000). This is because origin country or identity (otherness) were suggested as less important when arrivals' favourable traits are recognised (Kootstra 2016; Reeskens and van der Meer 2019). Descriptions of Syrians as illegitimate amplifies the deservingness gap with Ukrainians, additionally to anti‐Muslim bigotry (identity). Ukrainians were mentioned with greater reference to legitimacy, while official EU‐level statistics categorised most 2015 arrivals as illegal (Frontex 2015).

**FIGURE 2:**
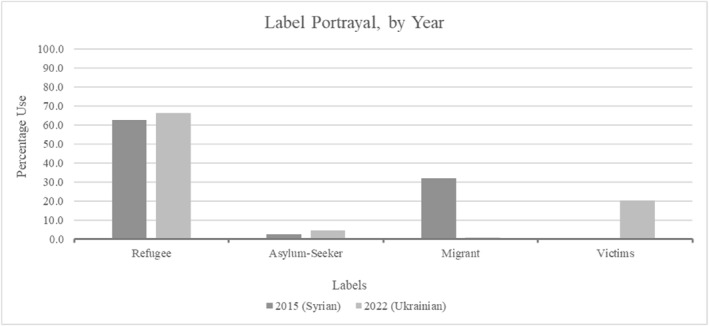
Labels used for arrivals.

This can be expanded to include labels referring to people, including refugee, migrant, asylum‐seeker or, as found regarding Ukrainians, victims (Figure 2). While Syrians and Ukrainians were largely labelled as refugees, the frequency ‘migrant’ was used for Syrians was notable. This underscores their lower deservingness or legitimacy than others, perceived as choosing to move with the necessary resources available (relating to control and need) following typical ‘economic migrant’ characterisations (Nielsen et al. 2020) or linked to criminality (smugglers).

Considering CARIN, UK government language suggested Ukrainians were well‐depicted, reflecting higher deservingness, especially when compared to the identity surrounding Syrians during 2015. It reveals how racialisation processes were more present for Syrians who were predominantly Muslim men, rather than women and families from traditionally Christian Ukraine (UNHCR 2015, 2022a). Greater assimilation could therefore be expected from Ukrainians, and less from Syrians subjected to negative racialisation by ministers; including through anti‐Muslim bigotry and lower assimilation expectations although both groups fled war as an otherwise ‘deserving’ reason (Hedegaard and Larsen 2022).

**FIGURE 3:**
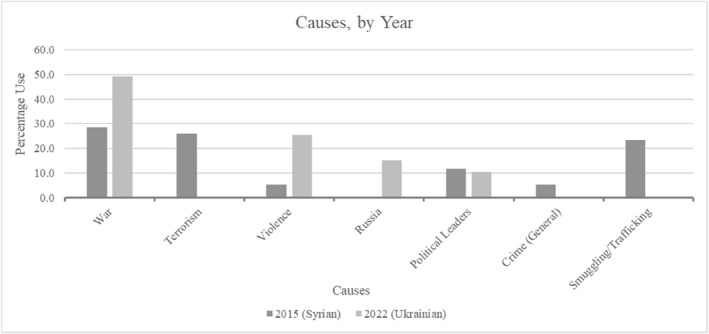
War, terrorism and crime as causes.

Similar was present regarding crisis causes with war, terrorism and criminality themes (Figure 3). War was the leading cause of mass displacement for both years. Violence in general was frequently mentioned for 2022, alongside direct mention of Putin as the political leader responsible; Assad the Syrian comparison. Russia was also identified as a cause in 2022, but not Syria, reflecting the conflicts' nature; one a Russian invasion, the other civil war, but again both conflicts perceivable as legitimate migration reasons (Hedegaard and Larsen 2022).

Most striking was the extent terrorism, ‘crime’ and smuggling as causes were present. Used only regarding Syria and the 2015 Migrant Crisis, this illegality and Islamist terror focus reinforces above deservingness and racialisation discussions. In brief, terrorism in Syria could spread and pose risks to Western nations with Muslims, often maintaining their traditions within host societies without further assimilation, possibly framed more as security threats given their identity (Abrahamsen 2005; Goldberg 2009; Huysmans and Buonfino 2008; Younis and Jadhav 2020).

This illegality, including the smuggling routes used, also contrasted contrast against Ukrainians' positive reception. Linkages could thus be made to greater un‐deservingness based on racialised identities and perceived illegitimacy. The relevant deservingness criterion becomes attitude crime‐related associations, presenting arrivals as illegally entering; reflecting bad behaviour used to construct negative, ‘othered’ portrayals of Syrians (Grove and Zwi 2006).

**FIGURE 4:**
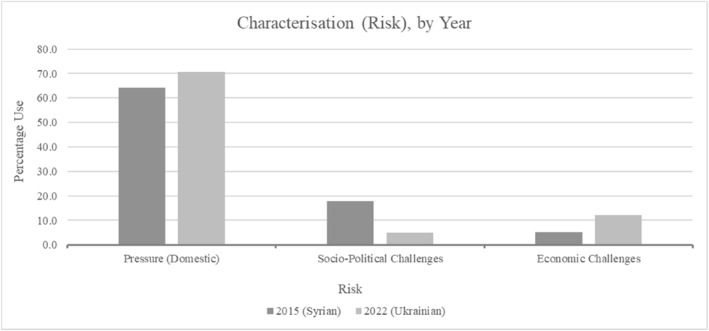
Event risks.

Socio‐political challenges can be considered additional pressures exerted on domestic systems (Figure 4). These relate to social cohesion and policy, reflecting low mixed embeddedness. Social cohesion and welfare system strain fall within Huysmans and Buonfino's (2008, 781) ‘politics of unease’ in UK government migration discussions understood to maintain a security‐related approach, albeit with greater subtlety. It speaks further to anti‐Muslim bigotry through racialised portrayals of Syrians and the crisis they fled. Ukrainian‐related social cohesion and identity concerns were absent. Resultantly, data again pointed towards the attitude criterion within 2015's data.

Socio‐political challenges signalled concerns around Muslim arrivals' impacts and perceived disruption. This related to mixed embeddedness and the non‐secular characterisation of Muslims within a secular society. Their lower mixed embeddedness compared to Ukrainians could thus be associated with negative behaviour (attitude) from Syrians concerning assimilation, coupled with anti‐Muslim bigotry. Less deservingness for Syrians was subsequently observed within UK minister language.

### 6.2 Reciprocity and Social Investment

**FIGURE 5:**
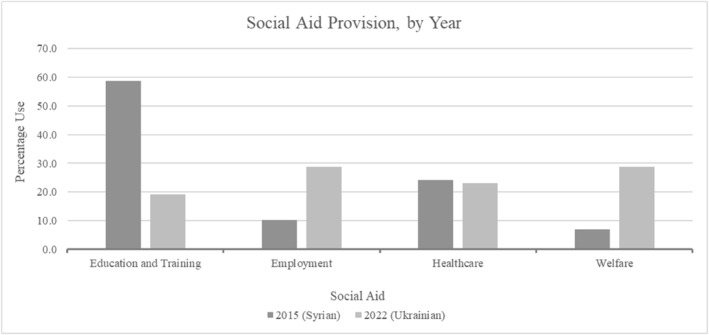
Provisions for arrivals.

Post‐arrival, focus centred on provisions that would be granted. This raised interesting points, particularly regarding deservingness' reciprocity notion. Syrians were expected to require training and education while Ukrainians were mentioned significantly more regarding direct labour market access (Figure 5). Moreover, Ukrainians were discussed as being granted welfare more than Syrians, incorporating benefits, social protection and childcare.

Regarding welfare, Ukrainians were seen to possess greater capabilities to reciprocate and thus be more deserving of this provision. This could be perceived according to their greater access and ability to work, contributing through work and taxes noted by others to increase deservingness (Abbas and Chrisp 2023; Nielsen et al. 2020), similarly to perceptions of arrivals' educational levels (Hedegaard and Larsen 2022). It could therefore be another indicator of higher Ukrainian deservingness which overcame, at least partially, welfare chauvinism within the UK.

An education focus could reflect perceptions around Syrians' ability to enter directly into work based on Syria's more challenging economic conditions (World Bank 2022), although Syrian and Ukrainian university graduate numbers were similar overall (UNHCR 2015, 2022a), with 38% of Syrian refugees holding university‐level education (Deloitte 2017), and Ukrainian graduates comprising 35% to 50% of Czechia‐ and Poland‐bound arrivals (Luděk et al. 2023). Consequently, a perceived requirement for greater support for Syrians compared to Ukrainians existed; the latter able to reciprocate in the present through higher employment prospects, the former potentially contributing later following social investment ideas (Heuer and Zimmermann 2020). This construction of Ukrainians as able to reciprocate immediately through work, while Syrians required further education before future social investment, mirrored greater public acceptance of Ukrainians through labour market participation found also in some EU nations, including Poland and Czechia (Letki et al. 2024; Luděk et al. 2023). This linked origin nations' economic development to reciprocity or social investment with language differences between 2022 (short‐term reciprocity through work) and 2015 (longer‐term social investment through education).

For education, less reference to Ukrainians suggested belief that provisions granted Ukrainians would be temporary. Combined with abilities to work directly on arrival and reciprocate benefits received, this may offer an understanding concerning why Ukrainians were officially portrayed as more deserving then Syrians. Figure 1 demonstrated this where Ukrainians were presented as going to return home, reflecting UK minister comments that they ‘do not want to be refugees’, ‘want to be Ukrainians living in Ukraine’ and ‘want to be able to go back and rebuild’. They were understood to be in the UK for the short‐term unlike resettled Syrians who needed education‐related investment and may not easily reciprocate. Consequently, UK ministerial language pointed towards continued welfare chauvinism interwoven with deservingness issues.

Ukrainians were welcomed and portrayed in a more positive light as deserving families and legal entrants able to reciprocate through work; all key CARIN elements (Van Oorschot 2000). However, this may change should Ukrainians remain in the UK beyond the shorter‐term stays expected, and recent research corroborates this by suggesting that public support for Ukrainian refugees has already reduced (Moise et al. 2024).

It may further reflect the greater acceptability of in‐work rather than out‐of‐work benefits in the UK (Schweyher et al. 2019), including how receipt is subject to additional conditionality since the Conservatives' welfare reforms from 2012 that require in‐work benefit recipients to actively seek ways of increasing earnings to reduce benefits needs (Abbas and Chrisp 2023).

However, human capital and demographics were prominent considerations that reflected racialisation. In previous studies, families fulfilled the identity, need and social investment criteria the most (Heuer and Zimmermann 2020). Ukrainians possessed more family demographic traits, including under‐18s, than Syrians. Accordingly, human capital investment should be higher in Ukraine‐related discussions, yet racialisation regarding human capital is clearly positive for Ukrainians but not Syrians given the demographic and educational characteristics of both groups.

### 6.3 Need and Control

**FIGURE 6:**
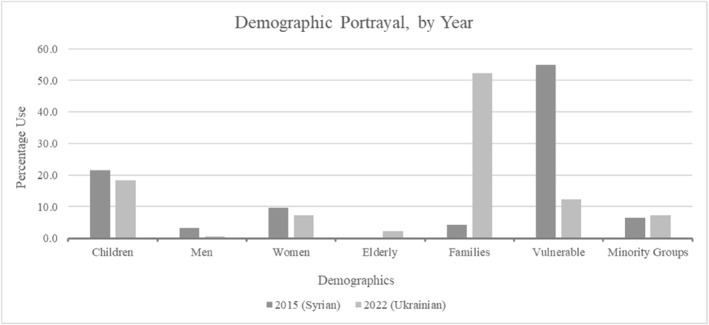
Demographic descriptions of arrivals.

Lastly, need and control can be considered regarding ministers' reference to arrivals' demographic characteristics (Figure 6), and these criteria have been found to be closely interrelated (Nielsen et al. 2020). There was roughly equal reference to children, women and minority groups between 2015 and 2022. The starkest differences were uncovered regarding arrivals' vulnerability, higher in 2015, and as being a family. Men were mentioned more for 2015, and elderly for 2022; the latter a generally more deserving group (Baekgaard et al. 2023).

The family code was most common for Ukrainians and this has repercussions following CARIN. Families have been considered the most deserving group among arrivals since their condition implies increased need and reduced control over migration causes (Laenen et al. 2019; Heuer and Zimmermann 2020). Accordingly, Ukrainian refugees may have been portrayed in a more positive light which rendered their identity as legitimate to receive support. Recall also that most Ukrainian arrivals travelled with close family; over 30% being under‐18s (Home Office 2022; UNHCR 2022a). Need, defined through deservingness, could thus be fulfilled more by Ukrainians.

Moreover, data suggested that portrayal as vulnerable and fleeing conflict does not necessarily equate to higher perceived deservingness. This becomes additionally interesting and relevant when considering the control criteria. Most Ukrainian refugees were women in 2022, but those in 2015 predominantly Syrian men with 2 and 5 times more men than women in the EU and UK, respectively (Eurostat 2025; Supporting Information S1: Appendix Tables AI–AIV). The latter could be understood in a negative light through the nexus of racialisation and control. This related to expectations that men should participate in and show responsibility (control) for conflict. This was seen with Ukraine which introduced conscription following Russia's invasion. Accordingly, Ukraine family and sponsorship scheme arrivals to the UK were 65% women and 35% men (Home Office 2023; Supporting Information S1: Appendix Table AIV).

Consequently, Syrian men could have been considered less deserving following the control criteria since they fled Syria for Europe. This perceived deservingness could also be associated with the preference for immigrant women based on colonialism which particularly characterised men as less reliable and more violent (Goldberg 2009). A sex‐related element thus becomes identifiable whereby women, families and, by extension, Ukrainians, were more deserving. Moreover, within UK parliamentary debates, references to ‘vulnerability’ regarding Muslims may be associated with the vulnerability to become radicalised through counter‐terror perspectives, especially for men (Rodrigo‐Jusué 2023). This again reduces Syrian and Muslim deservingness in the UK.

One final important area concerns what actions government ministers considered to prevent refugee travel in the first instance. Striking was how discussions about helping people stay inside their origin country or region, and so outside the UK, was only present in 2015. This related to reducing push factors like poverty and instability, or pull factors including welfare entitlements. Put differently, greater efforts to avoid Syrians' arrival was espoused through ministers' statements without the same language for Ukrainians.

These differences were consequently reflected in adopted policies. This includes the UK's settlement support for Syrians remaining in the region (Jordan, Turkey, Iraq) combined with a refugee cap and more negative welfare deservingness perceptions (Home Office 2017). Contrastingly, Ukraine schemes were without limit (261,000 visas granted up to 2024 based on both family and sponsorship schemes) and without the welfare and labour market access restrictions on Syrians before refugee status confirmation (Cuibus et al. 2024; Home Office 2024).

## 7 Conclusion

According to deservingness criteria, this research's question concerning racialisation and welfare chauvinism was answered. Firstly, welfare deservingness regarding identity and attitude was racialised through a language of family and victimhood for Ukrainians, but crime and illegitimacy for Syrians. Resultantly, governmental language indicated how common deservingness culture in the UK could be racialised based on anti‐Muslim bigotry with Muslim men arriving from conflict zones construed as less deserving and more problematic language related to CARIN (Van Oorschot 2006). While it should be analysed further, this suggested that the expected challenges posed to UK society from Syrian refugees were socio‐political based on the nation's deeper secular and colonial roots, combined with counter‐terrorism architecture emphasising Islamist radicalisation, including of those labelled ‘vulnerable’ (Rodrigo‐Jusué 2023; Younis and Jadhav 2020).

Economic challenges were the possible strains discussed concerning 2022, remaining in‐keeping with welfare support to Ukrainians. The most frequent description of Ukrainians was as families; the most deserving group (Heuer and Zimmermann 2020). This could reflect how more women refugees were found unlike in 2015 and demonstrates Ukrainians' stronger deservingness, especially alongside this article's other findings.

Does this racialisation consequently pose counteractive benefits for Ukrainians by reducing welfare chauvinism? Reciprocity and social investment deservingness criteria held relevant implications here. Refugees' current contribution was assumed based on relatively higher origin country economic development and educational services for Ukrainians than Syrians. As expected, employment support was mentioned more for Ukrainians while much higher educational and training support was found concerning Syrians. This result supported ideas regarding current and future contributions from Ukrainians and Syrians, respectively. However, levels of university education were similar and the population under working age was larger among Ukrainians (Deloitte 2017; Home Office 2022; UNHCR 2022a).

Accordingly, in UK government statements the different racialisation towards both groups cast a shadow over actual demographic compositions that would have alternatively suggested that the larger underaged population from Ukraine would require more human capital investment, while the high number of Syrian adults possessing university education would be able to immediately participate in the labour market. Subsequently, social investment should be mentioned more for Ukrainians than Syrians based on demographics. Therefore, greater mention of welfare benefits for Ukrainians reflects not only the deservingness of family but also racialisation based on higher expectations around their work‐based contributions as refugees well‐prepared to be assimilated quickly but, at the same time, temporarily.

Ultimately, Ukrainians were better welcomed in UK ministerial language, but this neither decreased welfare chauvinism nor indicated greater White preferences. They were perceived as short‐term refugees who could contribute to UK societies if well‐supported in the labour market; deserving welfare benefits within a clear reciprocal relationship, with in‐work welfare benefits already subject to a strict conditionality for receipt. Therefore, they were assumed to be returning to their origin country once the war concludes. This was clearly shown in Figures 1 and 2 with Syrians regarded more as migrants who could not or will not return to Syria, whereas Ukrainians were expected to settle less in the UK. Consequently, Ukrainian family scheme visa holders were required to extend their visas from early 2025 since they were limited to three years' validity, with this possible through the new Ukraine Extension Permission Scheme or sponsorship scheme for up to a maximum of 18 additional months (Cuibus et al. 2024; Home Office 2024). Comparably favourable social benefits for Ukrainians could therefore be attributed to work‐benefit notions rather than reduced chauvinism based on a different racialisation, and fostering greater economic opportunities for refugees in host nations may not result in longer‐term acceptance as others have suggested (Kaim et al. 2024; Letki et al. 2024).

Therefore, these perceptions could remain open to reconsideration as the Russia–Ukraine war lasts longer than anticipated. Otherwise, without clear policy guidance and public consensus, chauvinistic welfare arguments could still grow and Ukrainians who try to settle in the UK may face potential discrimination as their perceived deservingness reduces. A longer‐term perspective around refugee arrival and integration for Syrians and Ukrainians requires timely development to addresses continued welfare chauvinism.

Future research should focus on investigating policy practice around Ukrainian refugees as the war continues and, eventually, ends, to explore dynamics between UK ministers' expressed perspectives on Ukrainians and how this develops in line with the deservingness insights provided by the present study. This would be important work against the backdrop of welfare chauvinism in the UK and may usefully introduce a cross‐country comparative lens to uncover trends in other European host nations, like Poland and Czechia where Ukrainians were also accepted more willingly than Syrians were (Luděk et al. 2023).

These analyses could consider the language used by opposition parties through speeches and Parliamentary debates to identify similarities and differences in the perspectives expressed by this wider political actors (Huysmans and Buonfino 2008; Rodrigo‐Jusué 2023), including what they could mean for how refugees of different origins may be treated in practice. For example, in 2015 the opposition Labour Party challenged the UK government on its 20,000‐Syrian VPRS cap, arguing for an uncapped scheme like that later adopted for Ukrainians, yet language around demographics, smuggling gangs and similar themes remained shared (Cooper 2015; Hansard 2015).

The final suggestion is the fair presentations of official statistics given how UK statistics about Syrians' are not as detailed as those for Ukrainians. Pre‐Brexit, sex‐disaggregated UK data was available through Eurostat for Syrians whereas official UK figures from 2020 do not provide this data, unlike with Ukrainians (see Supporting Information S1). Furthermore, intersectional identities including education, age and sex were not specified for both refugee groups, with only Ukrainians' age and sex provided.

Standardised and transparent data for refugees irrespective of ethnicity is important because information gaps could further amplify chauvinism and negative deservingness perceptions towards certain minorities, including contributions to host societies through employment (Calo et al. 2024). Accordingly, fair data collection that reveals intersecting demographic information is required to help understand and alleviate prejudices while informing impactful policies supporting refugee integration under a long‐term perspective.

## Conflicts of Interest

The authors declare no conflicts of interest.

## Supporting information

Supporting Information S1

## Data Availability

Research data are not shared.
