# Supplementary material for: Ukrainian Refugees and Welfare Deservingness: A Comparative Study of UK Government Discussions Around the 2022 Ukraine Conflict and 2015 Migrant Crisis
Source: Br J Sociol. 2025 Apr 27;76(4):800–13. doi: 10.1111/1468-4446.13219 (PMC12412094; doi:10.1111/1468-4446.13219)

**Appendix: Asylum Applications, UK and EU**

Table AI: Syrian asylum application by sex in the EU and UK from 2015.

| **Syrian men asylum applications** | | | | | | | | | |
| --- | --- | --- | --- | --- | --- | --- | --- | --- | --- |
| Year | 2015 | 2016 | 2017 | 2018 | 2019 | 2020 | 2021 | 2022 | 2023 |
| EU | 259,285 | 207,490 | 55,215 | 43,595 | 41,845 | 42,845 | 81,000 | 103,430 | 143,960 |
| UK | 2,350 | 1,225 | 500 | 625 | 1,000 | n/a | n/a | n/a | n/a |
| **Syrian women asylum applications** | | | | | | | | | |
| Year | 2015 | 2016 | 2017 | 2018 | 2019 | 2020 | 2021 | 2022 | 2023 |
| EU | 105,955 | 129,610 | 48,980 | 39,705 | 34,410 | 25,730 | 35,025 | 32,015 | 42,525 |
| UK | 450 | 345 | 290 | 300 | 400 | n/a | n/a | n/a | n/a |

*Source*: Eurostat (2025), authors’ re-organisation.

Note: EU denotes the European Union, including 27 countries from 2020 and excluding the UK from 2019. UK-specific figures are not provided from 2019 due to Brexit and subsequent changes to data collection and reporting.

Table AII: Syrian asylum application in the UK from 2020.

| Year | 2020 | 2021 | 2022 | 2023 |
| --- | --- | --- | --- | --- |
| Applications | 1,482 | 3,389 | 3,885 | 3,132 |

*Source*: Home Office (2024) *Asylum and Resettlement Summary Tables, Year Ending September 2024 (Asy_01c)*, authors’ re-organisation.

Note: No sex-disaggregated data is provided by the government.

Table AIII: Ukrainian asylum applications in the EU in 2022 and 2023.

| Year | 2022 | 2023 |
| --- | --- | --- |
| Applications by Men | 12,190 | 7,960 |
| Applications by Women | 14,830 | 5,610 |

*Source*: Eurostat (2025), authors’ re-organisation.

Note: EU denotes the European Union including 27 countries and no UK-specific data is available from 2019 due to Brexit.

Table AIV: Age and sex breakdown for those arriving in the UK on Ukraine Schemes from March 2022 to December 2023.

| **Ukraine Schemes (UK)** | **Under 18** | **18-64** | **65+** | **Total** |
| --- | --- | --- | --- | --- |
| **Family Scheme Total** | **14,100 (7%)** | **35,800 (18%)** | **6,400 (3%)** | **56,300 (29%)** |
| Men (subset of Family Scheme Total) | 7,200 (4%) | 11,800 (6%) | 2,200 (1%) | **21,100 (11%)** |
| Women (subset of Family Scheme Total) | 6,800 (3%) | 24,000 (12%) | 4,300 (2%) | **35,100 (18%)** |
| **Sponsorship Scheme Total** | **40,300 (20%)** | **96,100 (49%)** | **4,400 (2%)** | **140,800 (71%)** |
| Men (subset of Sponsorship Scheme Total) | 20,300 (10%) | 27,100 (14%) | 1,300 (1%) | **48,600 (25%)** |
| Women (subset of Sponsorship Scheme Total) | 20,000 (10%) | 69,000 (35%) | 3,100 (2%) | **92,200 (47%)** |
| **Total Men** | **27,500 (14%)** | **38,900 (20%)** | **3,400 (2%)** | **69,800 (35%)** |
| **Total Women** | **26,900 (14%)** | **93,000 (47%)** | **7,400 (4%)** | **127,300 (65%)** |
| **Combined Total** | **54,400 (28%)** | **131,800 (67%)** | **10,900 (6%)** | **197,100 (100%)** |

*Source*: Home Office (2023) *Ukraine Visa Schemes Summary Tables, Year Ending December 2023 (UVS_05)*, authors’ re-organisation.

**Appendix: EU Language Frequencies for Syrians (2015) and Ukrainians (2022)**

Data was collected from the ‘newsroom’ websites of the EU Commission and Parliament for the same timeframes and with search keywords and coding schema applied as found in the main manuscript for UK-only results. The sample of EU documents was *n*=77 (44 for 2015, 33 for 2022). Used comparatively, the broader EU language was consistent with UK findings of the main article in how they portrayed Syrians as more illegitimate and as being exploited, related with smuggling, socio-cultural challenges and greater requirements for investment in their education. Ukrainians, meanwhile, were exclusively referred to as victims by both UK and EU officials. Some differences emerged, including how EU officials discussed children more for Ukrainians compared to the UK, while UK ministers placed greater emphasis on families than the EU. The latter could reflect the Ukraine family scheme as a key UK policy response that was developed.

Figure AI: Arrivals’ status portrayals.


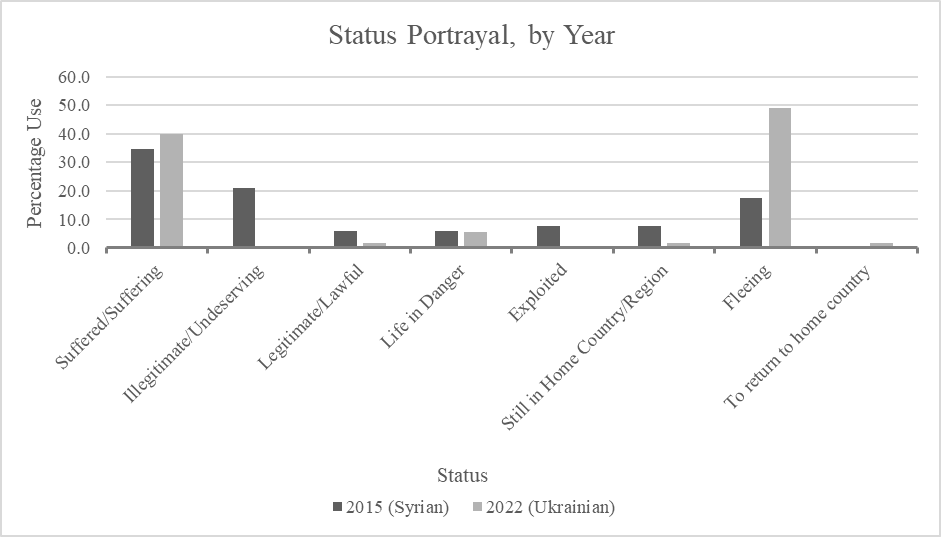


Figure AII: Labels used for arrivals.


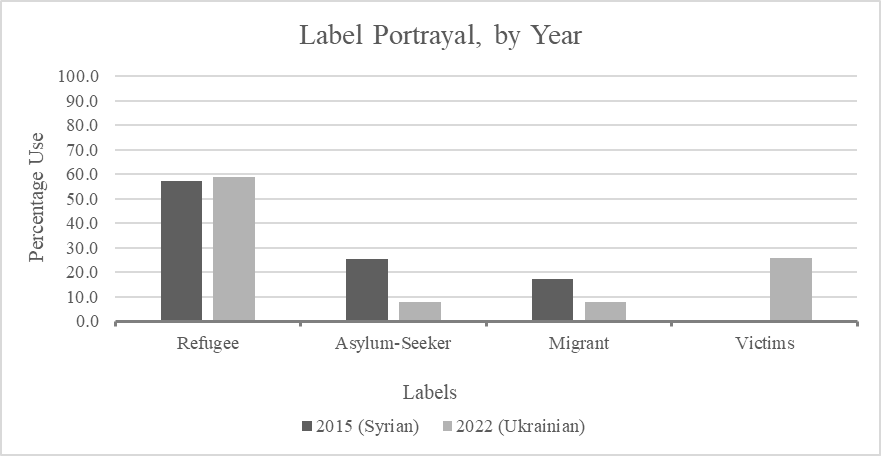


Figure AIII: War, terrorism and crime as causes.


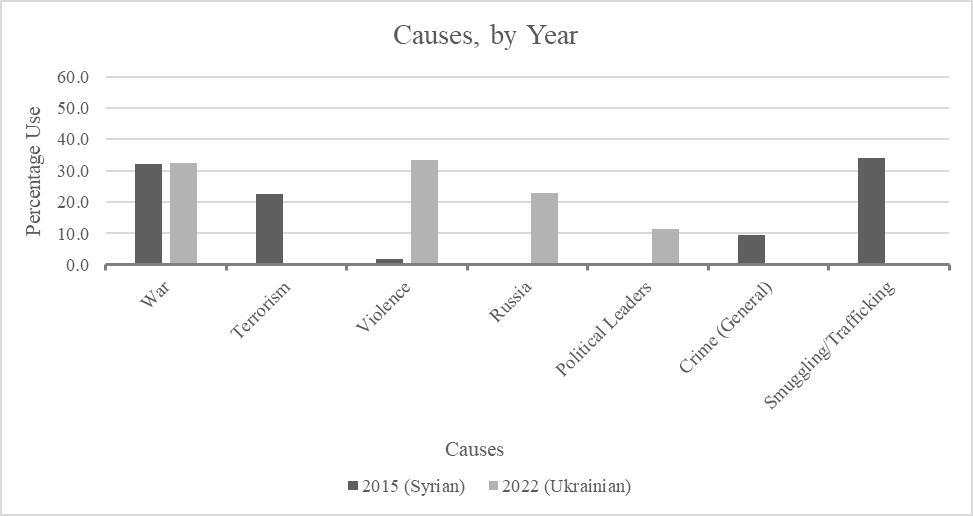


Figure AIV: Event risks.


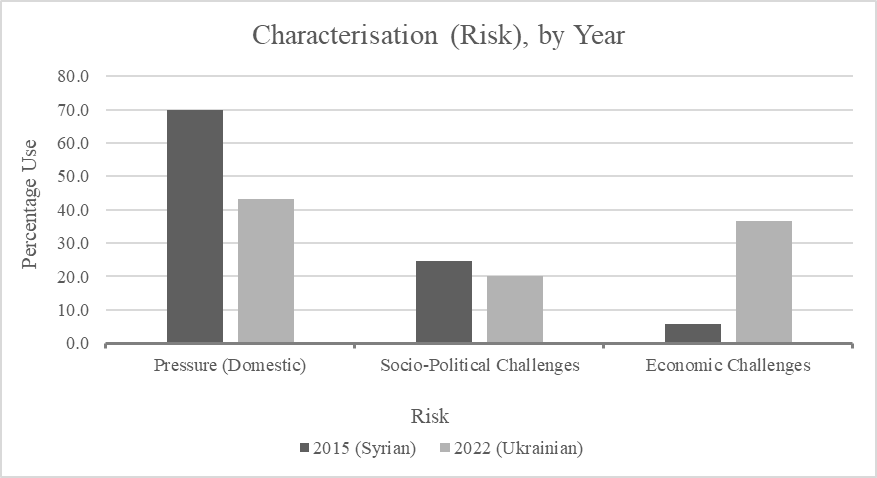


Figure AV: Provisions for arrivals.


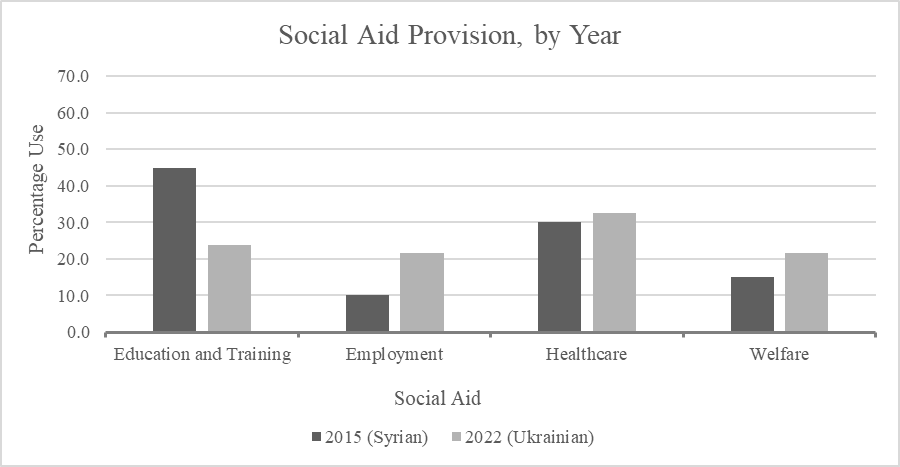


Figure AVI: Demographic descriptions of arrivals.


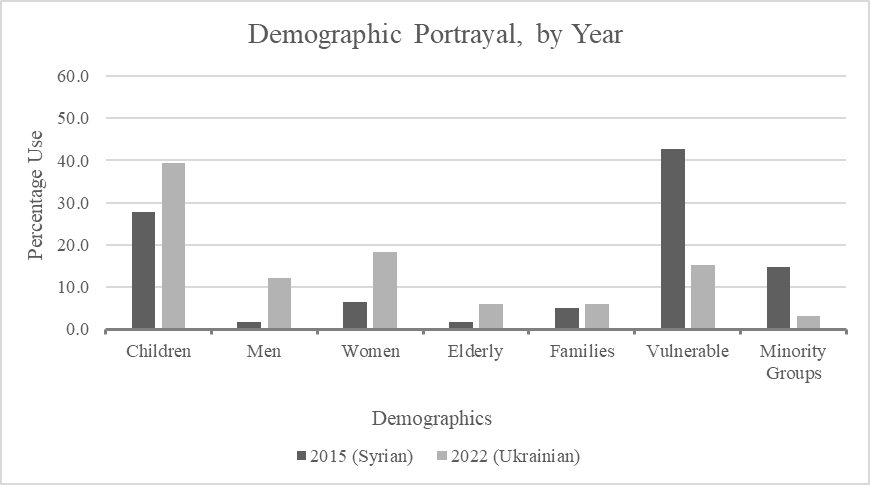

Supplement: Supplementary file 1 — Supporting Information S1 [file BJOS-76-800-s001.docx]
